# Income Inequality in Quality of Life among Rural Communities in Malaysia: A Case for Immediate Policy Consideration

**DOI:** 10.3390/ijerph17238731

**Published:** 2020-11-24

**Authors:** Govindamal Thangiah, Mas Ayu Said, Hazreen Abdul Majid, Daniel Reidpath, Tin Tin Su

**Affiliations:** 1 Centre for Population Health (CePH), Department of Social and Preventive Medicine, Faculty of Medicine, University of Malaya, Kuala Lumpur 50603, Malaysia; govindamal.t@gmail.com (G.T.); mas@ummc.edu.my (M.A.S.); hazreen@ummc.edu.my (H.A.M.); 2 South East Asia Community Observatory (SEACO) & Global Public Health, Jeffrey Cheah School of Medicine and Health Sciences, Monash University Malaysia, Subang Jaya 47500, Malaysia; Daniel.Reidpath@icddrb.org; 3 Department of Nutrition, Faculty of Public Health, Universitas Airlangga, Jawa Timur 60115, Indonesia; 4 International Centre for Diarrhoeal Disease Research, Dhaka 1212, Bangladesh

**Keywords:** income inequality, rural areas, quality of life, Malaysia, social determinants of health

## Abstract

Quality of life (QOL) is a proxy of health and social well-being. Hence, it is vital to assess QOL as it informs the strategies of policymakers to enhance the living conditions in communities. Rural areas in emerging economies are underserved in terms of modern facilities and technologies, which impact QOL. To address this, this study investigated whether income played a role in the QOL of rural residents within emerging economies using a large survey of Malaysian adults above 18 years old. The study extracted data from a sample of 18,607 respondents of a health and demographic surveillance system survey. A generalized linear model was used to estimate the impact of three income groups, the bottom 40%, middle 40% and top 20%, on perceived QOL, controlling for sociodemographic, chronic disease co-morbidities and mental health status. Results of the study showed a statistically significant association between income and the physical, psychological, social and environmental QOL domains. Using the bottom 40% as a reference category, the middle 40% and top 20% income groups showed a significant and positive association across the four domains of QOL. Hence, intervention programs are necessary to escalate the income levels of rural communities, especially the bottom 40%, to uplift perceived QOL among rural residents.

## 1. Introduction

Across the globe, rural populations are overwhelmingly poor [1,2,3,4], which significantly impedes overall life satisfaction. A staggering 580 million (79%) of the world’s poor reside in rural areas [2,5]. In developing countries, living in rural households increases the odds of being poor compared to urban counterparts [5,6]. Malaysia successfully attenuated its rural poverty rate to 1.0% in 2016, reduced from 58.7% in 1970 [7]. Although the average household income rose by twenty-fold from MYR 200 in 1970 to MYR 4359 in 2016 in rural areas, it has consistently remained lower than in urban areas over this period [7]. This is evidenced by the high share of the bottom 40% (B40) income group in rural areas (44.0%) [8], where a majority are heavily dependent on agricultural outputs which have low income returns [9].

Quality of life (QOL) often serves as a critical indicator of the government policies implemented and highlights health and psychosocial well-being issues in the population [10,11,12]. It encompasses a wide range of facets within the physical, social, environmental and psychological domains and is perceived as an efficient appraisal of one’s life satisfaction, desire, needs and aspirations within the context of one’s culture and values systems [13]. Thus, less fortunate rural dwellers, who are not only incapable of fulfilling their basic needs but also reside in less developed remote locations, most likely experience poor perceived QOL.

While the rich place great importance on freedom of choice related to life satisfaction, the poor are largely dependent on survival strategies to make ends meet. Hence, a rise in income levels provides the rural poor with basic necessities that would help them achieve a better QOL. Underpinning this, empirical studies from China [14], India [15], Malaysia [16,17,18] and Russia [19] showed a positive link between increasing income levels and QOL among rural dwellers. However, the findings by [16] were questionable as they used a small sample size that did not show generalizability and did not apply rigorous modeling methods to further substantiate the association between income and QOL. Moreover, Hassan et al. [17] used national level QOL measures and did not capture the individual level QOL that was applied in this study. Furthermore, using qualitative research design [17] only selected high-income female entrepreneurs as key participants, which could have led to a biased conclusion due to the lack of comparison with other income levels. Similarly, the findings by Idris et al. [18] were also inaccurate and not robust, owing to the small sample size and the lack of individual-level measurement of QOL.

On the contrary, Easterlin, in a seminal work, contended the exposition that higher income, on average, engendered a happier and more satisfied population until a certain income threshold, after which it diminished [20]. Echoing Easterlin’s findings, several other scholars also documented a curvilinear association between income and QOL across and within countries [21,22,23]. Besides, Sen argued that high income levels did not entirely reflect a good QOL and referred to Kerala and Sri Lanka as examples of achieving the desired QOL outcome despite low income status [24]. In support of Sen’s contention, a few other studies also showed the insignificant impact of income on QOL [25,26,27,28,29]. Thus, given the mixed findings and paucity of robust scholarly work on the association between income and perceived QOL in rural settings, this study seeks to investigate this association within the Malaysian context.

## 2. Materials and Methods

### 2.1. Study Site

The sample of this study was collected by the South East Asia Community Observatory (SEACO) team located in the district of Segamat, Johor state, Malaysia. SEACO is a health and demographic surveillance system (HDSS) center, which operates and conducts annual surveys in five out of eleven sub-districts of Segamat. It is a unique research platform in assessing population health and well-being of semi-urban and rural communities, which the majority of the Malaysian population belongs to. Segamat and its five sub-districts were chosen based on the strong pre-existing relationship between the Jeffrey Cheah School of Medicine and Health Sciences (JCSMHS) and the district, as well as state health administration, which was essential to conduct this research. Segamat has a marked ethnic breakdown that closely reflects the national proportions of Malays (60%), Chinese (23%) and Indians (7%), as well as equal gender composition (male: 49%; female: 51%) [30].

### 2.2. Study Design, Sample and Data Collection Process

A total sample of 25,512 respondents enrolled in this study survey. Of this number, 18,607 participants aged 18 years and above were drawn and used in the analysis of this study. The study was only based on a baseline cross-sectional survey collected in 2013. All trained enumerators and staff briefed participants about the objectives of the survey, and only participants who gave written consent were recruited and enrolled. Respondents were approached at their respective residences to gather information on their sociodemographic background (age, gender, education, employment status, income, marital status, ethnicity), health-related conditions (diagnosed chronic illnesses) and quality of life using standardized health data collection tools. This information was recorded directly into Android mobile devices and tablets with survey forms designed in Open Data Kit (ODK). Data recorded on the tablets were then encrypted and uploaded to a secure server. The study was approved by the Monash University Human Research Ethics Committee (2013-3837-3646).

### 2.3. Outcome

#### QOL Assessment

Previous studies mostly applied either objective or subjective QOL indicators as an overall measure of QOL [31,32,33]. While early works have focused on evaluating objective QOL using quantifiable indicators, such as health care, education and more [18,34], subjective QOL measures individuals’ perception of life experiences [35]. A validated Malay version of the abbreviated World Health Organization Quality of Life (WHOQOL-BREF) self-reported questionnaire, which was tested as reliable and effective among Malaysians, was used to evaluate the perceived QOL [36]. WHOQOL-BREF instruments are valid, reliable and best applied in a diverse cross-cultural setting that is internationally comparable [37,38]. These items assess an individual’s life satisfaction and their perception of life from various aspects [38]. Moreover, its high acceptance among individuals worldwide minimizes refusal rates and missing data, which improves the accuracy in decision making processes and policy implementations [39].

The WHOQOL-BREF questionnaire consists of 26 instruments, of which 24 items are differentiated into four domains, namely physical health (seven items), psychological health (six items), social relationships (three items) and environment (eight items). Two other items, self-reported QOL and health satisfaction, are self-explanatory and are not assessed in this study. The items under each domain are listed in the Appendix A. Each item has a five-point Likert-scale response option ranging from 1 (very dissatisfied/very poor) to 5 (very satisfied/very good). The domain scores and their transformed linear scale between 0 and 100 are computed following a scoring guideline [13]. Higher scores suggest favorable perceived QOL environmental, social, psychological and physical domains.

### 2.4. Explanatory Variables

#### 2.4.1. Monthly Household Income

Monthly household income groups were characterized into three categories, namely, bottom 40% (B40), middle 40% (M40) and top 20% (T20), based on the income thresholds provided by the Department of Statistics Malaysia (DOSM) in 2014. The B40 income group was defined as individuals with monthly household income below RM 3860, the M40 income group were those with a monthly household income between RM 3860 and RM 8319 and the T20 income group were classified as those with monthly household income greater than RM 8319.

#### 2.4.2. Control Variables

Past studies have shown that demographic factors, such as age, gender, ethnicity and marital status, socioeconomic variables (education status, employment status), mental health status (mainly depression, stress and anxiety), diagnosed chronic diseases (stroke, heart disease, asthma, arthritis, urinary tract disease, kidney disease), impacts QOL [14,40,41]. Therefore, these variables were included in the analysis to control their influence on the association between income groups and perceived QOL. In doing so, the study achieved a more rigorous understanding of the impact of income groups on perceived QOL.

### 2.5. Statistical Analysis

The frequency of variables was recorded, and a chi-square test was conducted to identify the presence of significant bivariate associations between the predictor variables and income groups. Next, a generalized linear model (GLMz) method was applied to investigate the association between income groups and perceived QOL. GLMz was used because the residual of outcome variables was non-normally distributed. In addition, the analysis was adjusted for the control variables mentioned above to eliminate the effect of potential confounders. A robust Huber–White sandwich estimator was also used to avoid heteroskedasticity issues, further contributing to the rigorousness of the model produced. A multicollinearity test based on the variation inflation factor (VIF) was performed to identify the existence of any collinearity issues among the predictor variables. The VIF values for all variables were less than 5.0, indicating the absence of collinearity. Since the missing values were less than 16.0% and did not pass the 50.0% threshold level, all variables were included in the study [42]. All analyses were performed using SPSS version 20, with a 5% and 10% significance level.

## 3. Results

Table 1 below presents the prevalence and descriptive statistics of the variables included in the study. The average age of the respondents in the sample is 47.6 years, with a standard deviation of 16.9 (mean ± SD = 47.6 ± 16.9). The sample largely comprises Malays (65.1%), married couples (70.0%) and B40 income groups (68.5%), which closely represents the characteristics of a semi-urban and rural population. Income distributions are often skewed, which is apparent in many past works [43,44,45]. Therefore, the use of mean as a measure of income is not reliable because it loses its power to produce accurate results [44]. Also, previous studies that use mean income often ignore the different impacts of demographic and socioeconomic variables on each income level [43]. Hence, income groups are better and more commonly used to examine income differentials [45]. Table 1 also shows high shares of respondents who do not suffer from self-reported depression (81.9%), stress (90.9%) and anxiety (77.9%) levels, as well as low rates of those diagnosed with heart disease (3.0%), stroke (0.9%), asthma (3.7%), kidney disease (0.8%), urinary tract disease (0.9%) and arthritis (9.2%).

In addition, the bivariate associations show highly significant results (0.1% significance level) between the demographic variables (marital status, gender and ethnicity), socioeconomic status (education, employment status), mental health (depression, stress and anxiety), all diagnosed chronic diseases except urinary tract and kidney diseases and the three income groups, B40, M40 and T20 (Table 2). High proportions of those aged 60 years and above, other ethnicities, widowers, females, and those who are unemployed and illiterate belong to the B40 income group (Table 2). The characteristics of individuals within the M40 income group include those who are less than 20 years old, Chinese, single, male, employed and tertiary educated (Table 2). The T20 income group consists of individuals between age 20–39, Indians, singles, men and those who are employed and tertiary educated (Table 2).

Table 3 shows the adjusted effects of income groups on the four different domains of QOL, environmental (Model I), physical (Model II), social (Model III) and psychological (Model IV). The results show that, relative to B40, both M40 and T20 income groups are significantly and positively associated with all four domains of QOL, adjusting for age, gender, ethnicity, marital status, employment status, education level, diagnosed chronic diseases and mental health status. However, those who are unemployed are negatively associated across all the domains compared to housewives/husbands, and this is significant after adjusting for control variables. Similarly, pensioners are also significantly and negatively associated with all domains except the social domain. On the contrary, self-employed individuals are positively linked with all domains except the environmental domain. Against housewives/husbands as the reference category, those with paid employment show a positive but weak association with all domains except for the physical domain. However, this association is only significant at a 10% significance level with the social domain.

In terms of education level, only those with secondary and primary education showed a significant positive association with the physical domain compared to illiterates. Against illiterates as the reference category, those with primary, secondary, tertiary and other education showed a highly significant positive association with the psychological and environmental domain. However, there was a significant association between education level and the social domain. All age groups relative to those 60 years and above showed a significant positive association on all four domains of QOL. Malay and Indian ethnic groups showed a significant positive association across all four domains compared to the Chinese group. Gender, however, did not show any statistically significant association across all four domains of QOL. Those diagnosed with chronic diseases, such as kidney disease, arthritis, stroke and asthma, were all statistically and negatively associated with all domains of QOL compared to those who were undiagnosed. Similarly, those who were severely or extremely severely depressed and with mild or moderate anxiety showed a statistically significant negative association across all four domains controlling for other potential influences of QOL.

## 4. Discussion

The evidence produced from this study clearly proved the income inequality of QOL among rural residents. The M40 and T20 income groups had a better QOL in all domains (physical, psychological, social and environmental) compared to the B40 community. This concurred with the findings of previous studies done on rural areas of Russia [19], China [14], India [15], Malaysia [16,17,18] and several other developing countries [46]. However, our outcomes differed from studies that concluded the insignificance of income in explaining QOL [28,29]. The lack of consensus between the two sets of studies is most likely elucidated by the subjective perception of whether income is adequate to satisfy one’s need, the relative income position compared to income of other individuals and adaptation to new situations as expectation level changes [47].

Juxtaposed against M40 and T20 income groups, the less advantaged population (B40) experiences a much slower income growth in rural areas [7]. This is believed to be caused by the under-investment in infrastructure and facilities, limited job opportunities, high dependency on declining agricultural output, and relatively low pay in exchange for labor-intensive work [48]. Income scarcity weakens the purchasing power of rural dwellers and their ability to make ends meet, which in turn leads to poor perceived QOL.

The lack of income also inhibits rural people from procuring health-related equipment, products and services, as well as gaining health-related knowledge that would otherwise boost their physical mobility, fitness and health status [49]. This sheds light on the findings of this study, which showed a significant positive association between M40 and T20 income groups and the physical domain of QOL compared to the B40 income group. Likewise, other studies on rural areas in Kerala, India [15] and the Netherlands [50] have also arrived at the same conclusion. However, failure to include other major influences on QOL, such as chronic disease, employment status and ethnicity, questions the robustness of findings from the study in the Netherlands.

Findings on the positive association between the M40 and T20 income groups and environmental domain in this paper are most likely explained by their strong financial capacity that enables them to live in high-quality housing areas, which are quiet, safe, less polluted, with high access to green and open spaces as well as infrastructure [51,52]. In contrast, the outcome of a study on Australia showed that men with high socioeconomic status (SES) were less satisfied with certain aspects of the environmental domain [28] due to the pressure of their surroundings, a circumstance that increased one’s income level by comparing their income with that of the better-offs despite having strong financial security [53].

Moreover, the advantages of being exposed to green environments and fresh air, which include the promotion of good emotional health among the rich [51], helps substantiate the results of the significant positive association between M40 and T20 income groups and the psychological domain of QOL in this study. Also, those in the rural B40 group, at the bottom of the social ladder, often experience stress and anxiety problems when their income status is compared to those at higher rungs of the ladder, thus resulting in low scores in the psychological domain [46]. Growing concerns over their income position relative to better-offs often invokes negative feelings and emotions, such as envy, shame, guilt, anger, insecurity, social isolation and more [54,55,56], which negatively impacts the psychological domain of QOL [46]. Although an increase in income has been proven to enhance the emotional well-being of the poor, a study has shown that it does not have the same effect among the rich [57]. This explains the changes in life expectancy among the rich as income level rises [57].

While the results of the study showed a significant negative association between unemployed individuals and all four domains of QOL, paid-employees, the self-employed and others were positively associated across all domains of QOL. This is supported by [58], which concludes that unemployment leads to increased family stress [59] and poorer life satisfaction levels [60] compared to the employed. The lack of job security and scarcity of formal employment in rural areas hinders rural dwellers from the protection of severance payments and unemployment benefits available for those in formal employment, which can lead to severe economic hardship and food insecurity for the worker and those family members supported by this income.

Our results on the significant positive association between M40 and T20 income groups and the social domain relative to the B40 income groups in rural areas could be elucidated by the high rural exodus, especially among low-income earners in the United States [61]. A similar phenomenon was also observed in rural Malaysia [62]. This was accompanied by a declination in rural population growth [48], and engendered the deterioration of social institutions and increasing numbers of single, elderly populations in rural areas [63]. 

Interestingly, the findings of this study also showed no statistical significance between gender and the four domains of QOL, which was consistent with the findings of several other studies. Corroborating this, a meta-analysis of 146 studies showed negligible effects of gender on well-being, where it only accounted for less than 1% of the well-being scores [58], which also paralleled the findings of a cross-country study [58]. The lack of statistical significance could be a consequence of the increasing awareness of gender equality, which provides equal roles and opportunities to both males and females in the decision making process [64]. However, this negated the results of other studies that concluded a better QOL among men than females in rural areas. The outcome from this study also showed a better perceived QOL across all domains among younger residents (age 18 to 59 years) than the elderly (60 years and above), which was consistent with the results of previous works [41,50,65]. The deteriorating health conditions, including mental health, limited mobility and age-related prejudice, most likely explains the poor psychological, physical, social and environmental domains of QOL among the elderly population [41,64,66].

## 5. Conclusions

Overall, the M40 and T20 income groups enjoy a better perceived QOL than the B40 in rural areas. Hence, it is imperative to uplift the QOL of the B40 population in rural areas to help contribute to the country’s economic growth and raise its status to a developed nation. A better QOL also ensures equitable opportunities for all segments of the population, including the B40 rural households. With this, the B40 population will not be left behind in participating and benefiting from national development and prosperity. Therefore, the findings support the case for introducing intervention programs, such as entrepreneur development activities, and the provision of infrastructure and services, including roads, broadband, Internet access, e-commerce, telecommunications, education and more in rural areas. These, in turn, will create job opportunities and elevate the income levels of the B40 community, which enhances their QOL in rural areas. Furthermore, findings from this study can also inform policymakers to continuously monitor and implement intervention programs needed to increase the QOL among B40 income groups in rural areas.

## Figures and Tables

**Table 1:** Sample characteristics of rural communities (*n* = 18,607).

Characteristics By	Frequency, *n* (%)	Mean ± SD	Minimum	Maximum
**Demographic**				
Age	18,607 (100.0)	47.6 ± 16.9	18	98
Ethnicity				
Malay	12,113 (65.1)			
Indian	1831 (9.8)			
Chinese	4181 (22.5)			
Others	436 (2.3)			
Total	18,561 (99.7)			
Gender				
Female	10,280 (55.2)			
Male	8323 (44.7)			
Total	18,603 (99.9)			
Marital Status				
Single	3468 (18.6)			
Married	13,026 (70)			
Separated/Divorced	329 (1.8)			
Widow	1610 (8.7)			
Others	168 (0.9)			
Total	18,601 (100)			
**Socioeconomic Status**				
Employment Status				
Housewife/Househusband	5326 (28.6)			
Not Working	2288 (12.3)			
Paid Employment	6385 (34.3)			
Pensioners/Pensions	997 (5.4)			
Self Employed	2878 (15.5)			
Others	653 (3.5)			
Total	18,527 (99.6)			
Income Groups				
B40	12,742 (68.5)	1740.35 ± 999.60	0	3850
M40	3449 (18.5)	5408.64 ± 1186.69	3880	8300
T20	620 (3.3)	9778.68 ± 960.83	8350	11,600
Total	16,811 (90.3)	2789.40 ± 2263.56	0	11,600
Education Status				
No Formal Education	497 (2.7)			
Primary	5696 (30.6)			
Secondary	9967 (53.6)			
Tertiary	1558 (8.4)			
Other	549 (3)			
Total	18,267 (98.3)			
**Mental Health Status**				
Depression				
None	15,241 (81.9)			
Mild/Moderate	2369 (12.7)			
Severe/Extremely Severe	850 (4.6)			
Total	18,460 (99.2)			
Stress				
None	17,037 (91.6)			
Mild/Moderate	1199 (6.4)			
Severe/Extremely Severe	343 (1.8)			
Total	18,579 (99.8)			
Anxiety				
None	14,490 (77.9)			
Mild/Moderate	2701 (14.5)			
Severe/Extremely Severe	1291 (6.9)			
Total	18,482 (99.3)			
**Diagnosed Chronic Diseases**				
Heart Disease				
No	17,387 (93.4)			
Yes	554 (3)			
Total	17,941 (96.4)			
Stroke				
No	17,811 (95.7)			
Yes	170 (0.9)			
Total	17,981 (96.6)			
Asthma				
No	17,321 (93.1)			
Yes	680 (3.7)			
Total	18,001 (96.8)			
Kidney Disease				
No	17,721 (95.2)			
Yes	140 (0.8)			
Total	17,861 (96)			
Urinary Tract Disease				
No	17,742 (95.4)			
Yes	169 (0.9)			
Total	17,911 (96.3)			
Arthritis				
No	16,186 (87)			
Yes	1718 (9.2)			
Total	17,904 (96.2)			
Quality Of Life Domains				
Physical	18,501 (99.4)	60.5 ± 11.8	0	100
Psychological	18,507 (99.5)	70.9 ± 12.6	12.50	100
Social	15,734 (84.6)	70.3 ± 14.0	0	100
Environmental	18,515 (99.5)	65.8 ± 12.9	0	100

Note: Total does not add up due to missing values, SD—standard deviation.

**Table 2:** Bivariate association between income groups and other predictor variables.

Variables	Income Groups						χ^2^	*p*-Value
	B40		M40		T20			
	*n*	%	*n*	%	*n*	%		
Age Groups							279.74	<0.001
Less than 20	511	71.2	186	25.9	21	2.9		
20–39	3424	69.5	1245	25.3	261	5.3		
40–59	5147	75.8	1420	20.9	221	3.3		
60 and above	3660	83.7	598	13.7	117	2.7		
Ethnicity							57.2	<0.001
Malay	8761	77.0	2219	19.5	399	3.5		
Chinese	1147	72.4	382	24.1	56	3.5		
Indians	2508	72.7	796	23.1	148	4.3		
Others	304	84.9	49	13.7	5	1.4		
Marital Status							269.9	<0.001
Single	2076	66.9	858	27.6	171	5.5		
Married	9014	76.4	2379	20.2	408	3.5		
Separated/Divorced	257	83.2	40	12.9	12	3.9		
Widow	1272	87.5	156	10.7	25	1.7		
Other	123	86.6	15	10.6	4	2.8		
Gender							40.5	<0.001
Male	5537	73.6	1652	22.0	330	4.4		
Female	7205	77.5	1797	19.3	290	3.1		
Employment Status							793.7	<0.001
Housewife/House Husband	4087	84.0	679	14.0	98	2.0		
Not Working	1757	85.1	278	13.5	30	1.5		
Paid Employment	3595	63.7	1703	30.2	347	6.1		
Pensioners	727	79.8	156	17.1	28	3.1		
Self Employed	2126	79.5	461	17.2	86	3.2		
Others	389	67.1	160	27.6	31	5.3		
Education Status							609.4	<0.001
No Education	381	89.6	38	8.9	6	1.4		
Primary	4309	83.8	728	14.2	106	2.1		
Secondary	6704	73.7	2025	22.3	372	4.1		
Tertiary	733	54.2	514	38.0	106	7.8		
Other	382	82.3	67	14.4	15	3.2		
Depression							189.5	<0.001
None	10,602	77.3	2663	19.4	453	3.3		
Mild/Moderate	1577	71.5	540	24.5	90	4.1		
Severe/Extremely Severe	443	59.5	225	30.2	77	10.3		
Stress							232.4	<0.001
None	11,899	77.2	3019	19.6	505	3.3		
Mild/Moderate	612	58.2	346	32.9	93	8.8		
Severe/Extremely Severe	208	66.9	81	26.0	22	7.1		
Anxiety							241.1	<0.001
None	10,003	77.1	2543	19.6	436	3.4		
Mild/Moderate	1969	76.9	516	20.1	77	3.0		
Severe/Extremely Severe	666	58.2	372	32.5	107	9.3		
Heart Disease								
Yes	380	81.5	73	15.7	13	2.8	7.7	<0.05
No	11,996	76.0	3227	20.4	564	3.6		
Stroke								
Yes	121	83.4	21	14.5	3	2.1		
No	12,254	76.0	3299	20.5	575	3.6		
Asthma							13.2	<0.01
Yes	499	81.9	98	16.1	12	2.0		
No	11,884	75.8	3226	20.6	569	3.6		
Urinary Tract Disease							0.085	>0.10
Yes	99	76.7	26	20.2	4	3.1		
No	12,237	76.1	3274	20.4	572	3.6		
Kidney Disease							1.88	>0.10
Yes	78	71.6	25	22.9	6	5.5		
No	12,225	76.1	3267	20.3	563	3.5		
Arthritis							19.1	<0.01
Yes	1151	80.1	258	18.0	28	1.9		
No	11,177	75.7	3035	20.6	547	3.7		

Note: χ^2^—Chi-square value, B40—bottom 40% income group, M40—middle 40% income group, T20—top 20% income group. B40 income group are individuals with monthly household income below RM 3860, M40 income group are those with monthly household income between RM 3860 and RM 8319 and T20 income group are classified as those with monthly household income greater than RM 8319.

**Table 3:** Fitted estimates by means of generalized linear model.

Variable	QOL Domains							
	Model 1 Physical		Model 2 Psychological		Model 3 Social		Model 4 Environmental	
	β	S.E.	β	S.E.	β	S.E.	β	S.E.
Age Group (years)								
Less than 20	1.62 ***	0.61	2.89 ***	0.66	3.24 ***	0.87	1.74 ***	0.67
20–39	1.29 ***	0.34	1.54 ***	0.36	2.92 ***	0.45	0.92 **	0.37
40–59	1.34 **	0.26	0.96 ***	0.27	1.84 ***	0.35	0.66 **	0.29
60 and above	(ref)	(ref)	(ref)	(ref)	(ref)	(ref)	(ref)	(ref)
Ethnicity								
Malay	1.79 ***	0.24	2.00 ***	0.28	2.00 ***	0.32	0.61 **	0.29
Indian	0.82 **	0.32	2.15 ***	0.37	0.95 **	0.41	0.73 *	0.38
Others	−0.77	0.58	−2.19 ***	0.62	−2.64 ***	0.79	−5.65 ***	0.66
Chinese	(ref)	(ref)	(ref)	(ref)	(ref)	(ref)	(ref)	(ref)
Gender								
Male	−0.02	0.24	0.04	0.27	0.07	0.31	0.12	0.27
Female	(ref)	(ref)	(ref)	(ref)	(ref)	(ref)	(ref)	(ref)
Marital Status								
Married	−0.76 **	0.31	0.50	0.34	2.45 ***	0.44	0.42	0.35
Separated/Divorced	−2.46 ***	0.65	−0.60	0.71	−0.83	1.14	−1.48 **	0.73
Widow	−2.43 ***	0.45	−0.92 *	0.48	−0.21	0.69	−1.57 ***	0.49
Others	−6.51 ***	0.53	0.78	0.66	−2.87	2.49	−2.44 ***	0.66
Single	(ref)	(ref)	(ref)	(ref)	(ref)	(ref)	(ref)	(ref)
Income Groups								
M40	2.59 ***	0.24	2.76 ***	0.26	2.38 ***	0.29	3.24 ***	0.26
T20	6.97 ***	0.54	3.90 ***	0.61	3.48 ***	0.73	5.97 ***	0.64
B40	(ref)	(ref)	(ref)	(ref)	(ref)	(ref)	(ref)	(ref)
Education Level								
Primary	2.68 ***	0.59	3.73 ***	0.69	0.69	0.78	4.04 ***	0.69
Secondary	2.22 ***	0.61	4.09 ***	0.71	1.15	0.80	4.29 ***	0.70
Tertiary	1.08	0.69	4.32 ***	0.80	0.75	0.91	4.52 ***	0.80
Other	0.70	0.74	3.15 ***	0.85	0.46	1.04	4.16 ***	0.88
No Formal Education	(ref)	(ref)	(ref)	(ref)	(ref)	(ref)	(ref)	(ref)
Employment Status								
Not Working	−1.23 ***	0.35	−1.27 ***	0.38	−1.69 ***	0.49	−2.25 ***	0.40
Paid Employment	1.43 ***	0.29	1.08 ***	0.31	0.72 *	0.37	0.77 **	0.32
Pensioners/Pensions	0.16	0.42	−1.80 ***	0.51	0.16	0.60	−1.31 **	0.51
Self Employed	1.15 ***	0.34	1.00 ***	0.34	1.19 ***	0.43	0.35	0.36
Others	3.13 ***	0.72	2.46 ***	0.75	2.37 **	1.01	2.42 ***	0.75
Housewives	(ref)	(ref)	(ref)	(ref)	(ref)	(ref)	(ref)	(ref)
Diagnosed Chronic Diseases								
Heart Disease								
Yes	−0.56	0.59	−1.87 ***	0.59	−2.11 ***	0.76	−0.95	0.61
No	(ref)	(ref)	(ref)	(ref)	(ref)	(ref)	(ref)	(ref)
Asthma								
Yes	−2.34 ***	0.46	−0.90 *	0.49	−1.79 ***	0.62	−1.72 ***	0.51
No	(ref)	(ref)	(ref)	(ref)	(ref)	(ref)	(ref)	(ref)
Stroke								
Yes	−6.55 ***	1.05	−4.72 ***	1.17	−5.34 ***	1.49	−4.54 ***	1.13
No	(ref)	(ref)	(ref)	(ref)	(ref)	(ref)	(ref)	(ref)
Arthritis								
Yes	−1.07 ***	0.34	−1.89 ***	0.37	−3.04 ***	0.45	−1.99 ***	0.37
No	(ref)	(ref)	(ref)	(ref)	(ref)	(ref)	(ref)	(ref)
Urinary Tract Disease								
Yes	−2.81 **	1.06	−2.06 *	1.15	−0.72	1.41	−1.67	1.25
No	(ref)	(ref)	(ref)	(ref)	(ref)	(ref)	(ref)	(ref)
Kidney Disease								
Yes	−3.34 ***	1.04	−3.28 ***	1.19	−6.39 ***	1.65	−3.90 ***	1.28
No	(ref)	(ref)	(ref)	(ref)	(ref)	(ref)	(ref)	(ref)
Depression								
Mild/moderate	0.64	0.42	−2.56 ***	0.46	−3.17 ***	0.55	0.05	0.46
Severe/Extremely Severe	−5.38 ***	1.35	−7.58 ***	1.44	−7.36 ***	1.61	−6.11 ***	1.37
None	(ref)	(ref)	(ref)	(ref)	(ref)	(ref)	(ref)	(ref)
Stress								
Moderate/Mild	0.77	0.75	0.01	0.80	−1.24	0.87	0.86	0.76
Severe/Extremely Severe	0.57	2.22	−5.06**	2.31	−2.14	2.54	0.17	2.12
None	(ref)	(ref)	(ref)	(ref)	(ref)	(ref)	(ref)	(ref)
Anxiety								
Mild/Moderate	0.96 ***	0.33	−3.29 ***	0.37	−1.88 ***	0.46	−2.86 ***	0.37
Severe/Extremely Severe	4.02 ***	0.91	−1.62	0.99	−3.16 ***	1.11	−0.83	0.95
None	(ref)	(ref)	(ref)	(ref)	(ref)	(ref)	(ref)	(ref)
Constant	55.51 ***	0.70	65.26 ***	0.81	66.17 ***	0.95	61.17 ***	0.82

Note: ***, **, * represent significance at 1%, 5% and 10% significance level, respectively; Models 1, 2, 3 and 4 represent the environmental, physical, social and psychological domains, respectively; ref—reference category, β—coefficient; S.E—standard error.

## Appendix A

**Table A1:** Characteristics of items within each quality of life (QOL) domain.

QOL Domains	Prevalence, *n*	Breakdown (%)	Cronbach’s Alpha
**Physical Domain**			
Pain			0.62
Very Often	4725	25.4	
Quite Often	6195	33.3	
Seldom	2234	12.0	
Never	307	1.6	
Total	18,497	99.4	
Energy			
Not At All	272	1.5	
A Little	852	4.6	
Moderately	6318	34.0	
Mostly	8485	45.6	
Completely	2580	13.9	
Total	18,507	99.5	
Sleep			
Very Dissatisfied	65	0.3	
Dissatisfied	640	3.4	
Neither Satisfied Nor Dissatisfied	3322	17.9	
Satisfied	10,990	59.1	
Very Satisfied	3461	18.6	
Total	18,478	99.3	
Mobility			
Very poor	147	0.8	
Poor	496	2.7	
Neither Poor Nor Good	4035	21.7	
Good	10,434	56.1	
Very Good	3386	18.2	
Total	18,498	99.4	
Activity			
Very Dissatisfied	63	0.3	
Dissatisfied	517	2.8	
Neither Satisfied Nor Dissatisfied	4336	23.3	
Satisfied	11,856	63.7	
Very Satisfied	1735	9.3	
Total	18,507	99.5	
Work			
Very Dissatisfied	58	0.3	
Dissatisfied	428	2.3	
Neither Satisfied Nor Dissatisfied	3557	19.1	
Satisfied	11,629	62.5	
Very Satisfied	2448	13.2	
Total	18,120	97.4	
Medication			
Always	4850	26.1	
Very Often	4380	23.5	
Quite Often	5917	31.8	
Seldom	2572	13.8	
Never	770	4.1	
Total	18,489	99.4	
**Psychological Domain**			0.73
Positive Feeling			
A Little	810	4.4	
A Moderate Amount	8751	47.0	
Very Much	7398	39.8	
An Extreme Amount	1290	6.9	
Total	18,508	99.5	
Think			
Not At All	192	1.0	
A Little	775	4.2	
A Moderate Amount	6667	35.8	
Very Much	8941	48.1	
Extremely	1923	10.3	
Total	18,498	99.4	
Esteem			
Very Dissatisfied	36	0.2	
Dissatisfied	297	1.6	
Neither Satisfied Nor Dissatisfied	2787	15.0	
Satisfied	12,151	65.3	
Very Satisfied	3199	17.2	
Total	18,470	99.3	
Body			
Not At All	204	1.1	
A Little	480	2.6	
Moderately	4508	24.2	
Mostly	7594	40.8	
Completely	5703	30.6	
Total	18,489	99.4	
Negative Feeling			
Always	129	0.7	
Very Often	826	4.4	
Quite Often	1861	10.0	
Seldom	9093	48.9	
Never	6547	35.2	
Total	18,456	99.2	
Spirituality			
Not At All	203	1.1	
A Little	476	2.6	
A Moderate Amount	5375	28.9	
Very Much	9011	48.4	
An Extreme Amount	3417	18.4	
Total	18,482	99.3	
**Social Domain**			0.80
Personal			
Very Dissatisfied	63	0.3	
Dissatisfied	468	2.5	
Neither Satisfied Nor Dissatisfied	3404	18.3	
Satisfied	10,294	55.3	
Very Satisfied	1527	8.2	
Total	15,756	84.7	
Sex			
Very Dissatisfied	80	0.4	
Dissatisfied	422	2.3	
Neither Satisfied Nor Dissatisfied	2632	14.1	
Satisfied	7929	42.6	
Very Satisfied	1823	9.8	
Total	12,886	69.3	
Support			
Very Dissatisfied	51	0.3	
Dissatisfied	408	2.2	
Neither Satisfied Nor Dissatisfied	4608	24.8	
Satisfied	11,872	63.8	
Very Satisfied	1480	8.0	
Total	18,419	99.0	
**Environmental Domain**			0.83
Safe			
Not At All	242	1.3	
A Little	739	4.0	
A Moderate Amount	6844	36.8	
Very Much	9545	51.3	
Extremely	1135	6.1	
Total	18,505	99.5	
Satisfied			
Very Dissatisfied	35	0.2	
Dissatisfied	365	2.0	
Neither Satisfied Nor Dissatisfied	2928	15.7	
Satisfied	13,024	70.0	
Very Satisfied	2157	11.6	
Total	18,509	99.5	
Finance			
Not At All	617	3.3	
A Little	1570	8.4	
Moderately	8741	47.0	
Mostly	5967	32.1	
Completely	1604	8.6	
Total	18,499	99.4	
Services			
Very Dissatisfied	41	0.2	
Dissatisfied	356	1.9	
Neither Satisfied Nor Dissatisfied	3628	19.5	
Satisfied	12,635	67.9	
Very Satisfied	1839	9.9	
Total	18,499	99.4	
Leisure			
Not At All	1306	7.0	
A Little	1961	10.5	
Moderately	7371	39.6	
Mostly	5934	31.9	
Completely	1925	10.3	
Total	18,497	99.4	
Physical Environment			
Not At All	172	0.9	
A Little	561	3.0	
A Moderate Amount	5883	31.6	
Very Much	9763	52.5	
Extremely	2112	11.4	
Total	18,491	99.4	
Transport			
Very Dissatisfied	47	0.3	
Dissatisfied	356	1.9	
Neither Satisfied Nor Dissatisfied	2763	14.8	
Satisfied	12,115	65.1	
Very Satisfied	3193	17.2	
Total	18,474	99.3	
Information			
Not At All	380	2.0	
A Little	1321	7.1	
Moderately	8452	45.4	
Mostly	6995	37.6	
Completely	1351	7.3	
Total	18,499	99.4	

## References

[1] Roser M., Ortiz-Ospina E. Global Extreme Poverty. https://ourworldindata.org/extreme-poverty.

[2] World Bank (2018). Poverty and Shared Prosperity 2018: Piecing Together the Poverty Puzzle.

[3] Abrar ul haq M., Jali M.R.M., Islam G.M.N. (2019). Household empowerment as the key to eradicate poverty incidence. Asian Soc. Work Policy Rev..

[4] IFAD Rural Development Report 2016 (2016). Fostering Inclusive Rural Transformation.

[5] UN General Assembly (74th sess. 2019–2020), Expert Group Meeting on “Eradicating Rural Poverty to Implement the 2030 Agenda for Sustainable Development”. https://www.un.org/development/desa/dspd/egm-rural-poverty.html.

[6] Bird K., McKay A., Shinyekwa I. (2010). Isolation and Poverty: The Relationship between Spatially Differentiated Access to Goods and Services and Poverty.

[7] Department of Statistics Malaysia (2016). Household Income and Basic Amenities Survey Report.

[8] Jayasooria D. (2016). Inclusive Development for Urban Poor & Bottom 40% Communities in Malaysia.

[9] Pemandu (2010). Economic Transformation Programme: A Roadmap for Malaysia.

[10] Diener E., Inglehart R., Tay L. (2013). Theory and validity of life satisfaction scales. Soc. Indic. Res..

[11] Meyer D.F., Dunga S.H. (2014). The determinants of life satisfaction in a low-income, poor community in South Africa. Mediterr. J. Soc. Sci..

[12] Pal A., Kumar U. (2005). Quality of Life (QOL) concept for the evaluation of societal development of rural community in West Bangal, India. Asia-Pac. J. Rural Dev..

[13] World Health Organization (1998). Programme on Mental Health: WHOQOL User Manual.

[14] Huang H., Liu S., Cui X., Zhang J., Wu H. (2018). Factors associated with quality of life among married women in rural China: A cross-sectional study. Qual. Life Res..

[15] Thadathil S., Jose R., Varghese S. (2015). Assessment of domain wise quality of life among elderly population using WHO-BREF scale and its determinants in a rural setting of Kerala. Int. J. Curr. Med. Appl. Sci..

[16] Sulaiman M., Hayrol A., Mohd S.O., Bahaman A.S., Asnarulkhadi A.S., Siti A.R. (2011). Factors affecting the quality of life among the rural community living along Pahang River and Muar River in Malaysia. Aust. J. Basic Appl. Sci..

[17] Hassan K., Ahmad Z., Arshad R. (2017). Does Increased in Incomes Improves Quality of Life of the Rural Low Income Households?. Int. J. Econ. Financ. Issues.

[18] Idris K., Shaffril H.A.M., Yassin S.M., Samah A.A., Hamzah A., Samah B.A. (2016). Quality of life in rural communities: Residents living near to Tembeling, Pahang and Muar Rivers, Malaysia. PLoS ONE.

[19] O’Brien D., Wegren S., Patsiorkovsky V. (2010). Sources of income, mental health and quality of life in rural russia. Eur. Asia Stud..

[20] Easterlin R.A. (1974). Does economic growth improve the human lot? Some empirical evidence. Nations and Households in Economic Growth.

[21] Cummins R.A. (2000). Personal income and subjective well-being: A review. J. Happiness Stud..

[22] Clark A.E., Frijters P., Shields M.A. (2008). Relative income, happiness, and utility: An explanation for the Easterlin paradox and other puzzles. J. Econ. Lit..

[23] Ed D., Biswas-Diener R. (2002). Will money increase subjective well-being? A literature review and guide to needed research. Soc. Indic. Res..

[24] Bloom D.E., Craig P.H., Malaney P.N. (2001). The Quality of Life in Rural Asia.

[25] Wyshak G. (2016). Income and subjective well-being: New insights from relatively healthy American women, ages 49–79. PLoS ONE.

[26] Sing M. (2009). The Quality of Life in Hong Kong. Soc. Indic. Res..

[27] Ahuvia A.C. (2002). Individualism/Collectivism and Cultures of Happiness: A Theoretical Conjecture on the Relationship between Consumption, Culture and Subjective Well-Being at the National Level. J. Happiness Stud..

[28] Brennan S.L., Williams L.J., Berk M., Pasco J.A. (2013). Socioeconomic status and quality of life in population-based Australian men: Data from the Geelong Osteoporosis Study. Aust. N. Z. J. Public Health.

[29] Ross N.A., Garner R., Bernier J., Feeny D.H., Kaplan M.S., McFarland B., Orpana H.M., Oderkirk J. (2012). Trajectories of health-related quality of life by socio-economic status in a nationally representative Canadian cohort. J. Epidemiol. Community Health.

[30] Partap U., Young E.H., Allotey P., Soyiri I.N., Jahan N., Komahan K., Devarajan N., Sandhu M.S., Reidpath D.D. (2017). HDSS profile: The South East Asia community observatory health and demographic surveillance system (SEACO HDSS). Int. J. Epidemiol..

[31] Prescott-Allen R. (2001). The Wellbeing of Nations.

[32] Vaishar A., Vidovićová L., Figueiredo E. (2018). Quality of Rural Life. Editorial 16 June 2018. Eur. Countrys..

[33] McCrea R., Marans R.W., Stimson R., Western J., Marans R.W., Stimson R. (2011). Subjective measurement of quality of life using primary data collection and the analysis of survey data. Investigating Quality of Urban Life.

[34] Moreno-Mínguez A., Martínez-Fernández L.C., Carrasco-Campos Á. (2016). Family policy indicators and well-being in Europe from an evolutionary perspective. Appl. Res. Qual. Life.

[35] Wojewódzka-Wiewiórska A., Kłoczko-Gajewska A., Sulewski P. (2020). Between the Social and Economic Dimensions of Sustainability in Rural Areas—In Search of Farmers’ Quality of Life. Sustainability.

[36] Hasanah C., Naing L., Rahman A. (2003). World Health Organization Quality of Life Assessment: Brief version in Bahasa Malaysia. Med. J. Malaysia.

[37] Gholami A., Jahromi L.M., Zarei E., Dehghan A. (2013). Application of WHOQOL-BREF in Measuring Quality of Life in Health-Care Staff. Int. J. Prev. Med..

[38] Skevington S.M., Lotfy M., O’Connell K. (2004). The World Health Organization’s WHOQOL-BREF quality of life assessment: Psychometric properties and results of the international field trial. A report from the WHOQOL group. Qual. Life Res..

[39] Skevington S.M., Epton T. (2018). How will the sustainable development goals deliver changes in well-being? A systematic review and meta-analysis to investigate whether WHOQOL-BREF scores respond to change. BMJ Glob. Health.

[40] Sengupta N.K., Osborne D., Houkamau C.A., Hoverd W.J., Wilson M.S., Halliday L., West-Newman T., Barlow F.K., Armstrong G., Robertson A. (2012). How much happiness does money buy? Income and subjective well-being in New Zealand. N. Z. J. Psychol..

[41] Bortolotto C.C., de Mola C.L., Tovo-Rodrigues L. (2018). Quality of life in adults from a rural area in Southern Brazil: A population-based study. J. Revista Saúde Pública.

[42] Hair J.F., Black W., Babin B., Anderson R. (2013). Multivariate Data Analysis. Always Learning.

[43] Goh K.L., Tey N.P. (2018). Personal income in Malaysia: Distribution and differentials. Econ. Bull..

[44] Donovan S.A. (2015). A Guide to Describing the Income Distribution.

[45] Greenville J., Pobke C., Rogers N. (2013). Trends in the Distribution of Income in Australia.

[46] Reyes-García V., Babigumira R., Pyhälä A., Wunder S., Zorondo-Rodríguez F., Angelsen A. (2016). Subjective wellbeing and income: Empirical patterns in the rural developing world. J. Happiness Stud..

[47] Ferrer-i-Carbonell A. (2005). Income and well-being: An empirical analysis of the comparison income effect. J. Public Econ..

[48] Economic Planning Unit (2010). Transforming Rural Areas to Uplift Wellbeing of Rural Communities.

[49] Phelan J.C., Link B.G., Tehranifar P. (2010). Social conditions as fundamental causes of health inequalities: Theory, evidence, and policy implications. J. Health Soc. Behav..

[50] Gobbens R.J., Remmen R. (2019). The effects of sociodemographic factors on quality of life among people aged 50 years or older are not unequivocal: Comparing SF-12, WHOQOL-BREF, and WHOQOL-OLD. Clin. Interv. Aging.

[51] Wong F.Y., Yang L., Yuen J.W.M., Chang K.K.P., Wong F.K.Y. (2018). Assessing quality of life using WHOQOL-BREF: A cross-sectional study on the association between quality of life and neighborhood environmental satisfaction, and the mediating effect of health-related behaviors. BMC Public Health.

[52] Science for Environment Policy (2016). Links between Noise and Air Pollution and Socioeconomic Status.

[53] Easterbrook G. (2003). The Progress Paradox: How Life Gets Better While People Feel Worse.

[54] Kawachi I., Subramanian S., Almeida-Filho N. (2002). A glossary for health inequalities. J. Epidemiol. Community Health.

[55] Marmot M., Wilkinson R. (2005). Social Determinants of Health.

[56] Gero K., Kondo K., Kondo N., Shirai K., Kawachi I. (2017). Associations of relative deprivation and income rank with depressive symptoms among older adults in Japan. Soc. Sci. Med..

[57] Kahneman D., Deaton A. (2010). High income improves evaluation of life but not emotional well-being. Proc. Natl. Acad. Sci. USA.

[58] Worach-Kardas H., Kostrzewski S. (2014). Quality of life and health state of long–term unemployed in older production age. Appl. Res. Qual. Life.

[59] Helliwell J.F., Putnam R.D. (2004). The social context of well–being. Philos. Trans. R. Soc. Lond. Ser. B Biol. Sci..

[60] Pohlan L. (2019). Unemployment and social exclusion. J. Econ. Behav. Organ..

[61] Johnson J.E., Taylor E.J. (2019). The long run health consequences of rural-urban migration. Quant. Econ..

[62] Jahan N.K., Allotey P., Arunachalam D., Yasin S., Soyiri I.N., Davey T.M., Reidpath D.D. (2014). The rural bite in population pyramids: What are the implications for responsiveness of health systems in middle income countries?. BMC Public Health.

[63] Baernholdt M., Yan G., Hinton I., Rose K., Mattos M. (2012). Quality of life in rural and urban adults 65 years and older: Findings from the National Health and Nutrition Examination survey. J. Rural Health.

[64] Lee K.H., Xu H., Wu B. (2020). Gender differences in quality of life among community-dwelling older adults in low-and middle-income countries: Results from the Study on global AGEing and adult health (SAGE). BMC Public Health.

[65] Xie J.F., Ding S.Q., Zhong Z.Q., Yi Q.F., Zeng S.N., Hu J.H., Zhou J.D. (2014). Mental health is the most important factor influencing quality of life in elderly left behind when families migrate out of rural China. Rev. Lat. Am. Enferm..

[66] Ran L., Jiang X., Li B., Kong H., Du M., Wang X., Yu H., Liu Q. (2017). Association among activities of daily living, instrumental activities of daily living and health-related quality of life in elderly Yi ethnic minority. BMC Geriatr..

